# The untapped potential of Games for Health in times of crises. A critical reflection

**DOI:** 10.3389/fpubh.2023.1140665

**Published:** 2023-02-28

**Authors:** Kevin Dadaczynski, Daniel Tolks, Kamil J. Wrona, Timothy Mc Call, Florian Fischer

**Affiliations:** ^1^Department of Health Sciences, Fulda University of Applied Sciences, Fulda, Germany; ^2^Center for Applied Health Sciences, Leuphana University Lüneburg, Lüneburg, Germany; ^3^Department Pedagogy, Nursing and Health, Medical School Hamburg, Hamburg, Germany; ^4^Department of Engineering and Mathematic, Bielefeld University of Applied Sciences, Bielefeld, Germany; ^5^Department of Health, Bielefeld University of Applied Sciences, Bielefeld, Germany; ^6^Medical School OWL, Bielefeld University, Bielefeld, Germany; ^7^Bavarian Research Center for Digital Health and Social Care, Kempten University of Applied Sciences, Kempten, Germany; ^8^Institute of Public Health, Charité–Universitätsmedizin Berlin, Berlin, Germany

**Keywords:** games for health, gamification, serious games, COVID-19, emergency situation, health inequalities

## Abstract

Given its promising role in public health to address hard to reach population groups, game-based interventions (i.e., Games for Health, G4H) have experienced growing interest in recent years. Therefore, it is surprising that they have played only a minor role during the COVID-19 pandemic. Hence, the aim of this paper is to reflect the opportunities and challenges of G4H especially during the pandemic but also with regard to future health crises. As commercial video games (i.e., those that primarily aim to entertain its users) were often used to deal with the containment measures during the COVID-19 pandemic, we call for greater cooperation with commercial game makers to distribute health-related messages *via* entertainment games. With regard to G4H we see a need to (i) strengthen the intervention theory underlying game-based applications, (ii) to enhance the appeal of games in order to maintain the interest of users in the long term, and (iii) to improve the evidence base using appropriate study designs. Finally, we argue for (iv) greater user involvement, both in terms of developing game-based approaches and as co-researchers in solving complex health problems.

## 1. Introduction

Health crises are usually associated with a high degree of uncertainty, not least because they can only be predicted to a limited extent and the data and knowledge base is highly dynamic. In the case of COVID-19, a novel Severe Acute Respiratory Syndrome Coronavirus-2 (SARS-CoV-2) was identified in December 2019 and has caused over 750 million confirmed cases and 6.8 million deaths worldwide to date (1). Most countries have initiated public health communication measures early on in the pandemic to provide up-to-date information on various aspects of the pandemic and to communicate recommendations (e.g., for individual protective behaviors) and containment measures (e.g., restrictions on public and private gatherings, closure of schools, stay at home orders). Mass media such as search engines, news portals, social media or apps were used as main sources of information provision and seeking (2), which also contributed to the infodemic and its negative consequences (e.g., spread of misinformation) (3, 4). However, research findings show disparities in the uptake of health-related information. Results from the Health Information National Trends Survey (HINTS) Germany revealed that non-seekers had a lower socioeconomic status, a lower perceived information-related self-efficacy, and a lower trust in information sources (5). Similarly, a higher educational status and a higher income were positively associated with web-based information seeking on COVID-19 in a cross-sectional survey among adults from Hong Kong (6). Such disparities in the search for and use of health-related information ultimately reinforce social inequalities, making less direct health communication channels (i.e., channels that communicate health-related messages more implicit and casual) necessary.

Entertainment education strategies that use popular digital media such as games, films or music to deliver health-related messages can be considered as indirect, i.e., less explicit health-communication channels that are characterized by fun and excitement. Among these, digital games in particular have attracted increasing interest in public health over the last few years, but have played only a minor role in the context of the COVID-19 pandemic. Next to commercial games that primarily aim to entertain its users, games for health (G4H) pursue explicitly defined health-related objectives (e.g., by providing information or setting impulses for behavioral change) and use gamification and serious games as main strategies. While gamification refers to the “use of game design elements in non-game contexts” [(7), p. 10], serious games are characterized to be intertwined with an educational approach by imparting knowledge or skills (8). Its foundation can be seen in motivation and communication theories such as the self-determination theory or the elaboration likelihood model that can be used to explain differences in information processing (9, 10). Existing reviews revealed positive small to moderate effects of gamification and serious games for health on behavioral determinants (e.g., knowledge) (11), health behaviors (e.g., physical activity, nutrition) (11, 12) or mental health (e.g., improvement of symptoms and wellbeing) (9, 13). In addition, van der Lubbe et al. (14) were able to identify objectives, rewards and story elements in their review as game mechanics most often used in interventions to empower vulnerable target groups for training skills, promoting behavior or transferring knowledge.

In light of previous findings and experiences, the following perspective aims at a critical reflection of digital games to counteract COVID-19. The focus is not to provide a review of available interventions [for a systematic overview see Kermavnar et al. (15)], but on the overarching question of whether the potential inherent entertainment games and G4H is currently being realized.

## 2. Commercial video games: Health hazard or worthwhile public health perspective?

Commercial games, i.e., video games that focus on entertainment and fun and usually require a large developmental budget, have long been debated in terms of their health risk potential despite mixed findings (16, 17). During the COVID-19 pandemic, an increase in game use has been reported for different age groups which was associated with negative consequences (e.g., reduced physical activity, sleeping problems) in some studies [e.g., (18, 19)]. However, other studies found positive associations with social interactions or mental health outcomes. Pearce and colleagues found that parents and their children used the game “Animal Crossing: New Horizons” as a tool for emotional and problem-focused coping or to foster social connections during the pandemic (20). Similar findings have been reported from an UK study among 781 participants aged 16 years or older. Qualitative analyses of the open-ended questions revealed that codes with positive impact on mental health were 10 times more frequent than codes with negative outcomes with escapisms, socializing with others and stress relief identified as the most common themes (21). Another study among players of the location-based game “Pokémon GO” and “Harry Potter: Wizards Unite” indicated that more than three quarter believed that playing these games during the pandemic were beneficial for their mental health (22). Next to the topics mentioned above, respondents also stressed that these location-based games have motivated them to exercise more frequently.

While these examples use existing video games to analyze their use and health effects, we are not aware of systematic collaborations between commercial game providers and public health researchers to use entertainment games to fight against COVID-19 (for an exception see section on user involvement). Supported by the World Health Organization, 18 game industry companies joined the #PlayApartTogether social media campaign in March 2020 to encourage players around the globe to practice physical distancing. As a means of distraction during the pandemic and accompanied containment measures (e.g., lockdowns) some companies provided their players with rewards through new features or game content. We anticipate great potential for public health research and practice in strengthening and expanding these forms of collaboration. Huge gaming companies have game products that enjoy great popularity among a large number of gamers. In the third quarter of 2022 alone, around 370 million users worldwide accessed the game titles of the three major game developers and publishers Activision, Blizzard and King (23). Research collaborations would allow to reach a large audience with public health messages embedded in game mechanics and content. As these games were regularly updated, it would be possible to adapt and disseminate health-related messages according to needs and the dynamically adapting base of evidence during times of crises. In addition, opportunities for health research could arise *via* the large user base, e.g., by approaching gamers to participate in studies *via* the game's communication channels.

## 3. Games for Health: More serious than fun?

Compared to entertainment games, G4H are often developed by or in cooperation with researchers. In their review, Kermavnar and Desmet (15) identified 43 digital serious games on health with focus on COVID-19. Most games address children, adolescents, and young adults, while older population groups or health care professionals were rarely approached. Interestingly, the vast majority of these games (*n* = 37) were single-player games, which contrasts with the previously mentioned entertainment games, that were explicitly used to connect with others particularly in times of lockdown and self-isolation. With regard to game type, quiz games and simulation games seemed to be most prominent. Just to give three examples: Developed by a research team from Brazil, the mobile application “COVID-19–Did You Know?” aims at promoting knowledge about COVID-19 through six topics with a total of 49 of true or false questions and corresponding information (24). In turn, in the simulation game “Plague Inc: The Cure” players have to control the global pandemic response, e.g., by tracking the spread of the outbreak, implement measures such as contact tracing (25). Moreover, “Antidote COVID-19” uses the successful tower defense game genre, where players need to help the human immune system to fight against SARS-CoV-2 while learning about the immune system and pathogens (26).

Although the extensive efforts of these and all other game developers are greatly to be appreciated, a number of challenges remain, some of which already existed before the COVID-19 pandemic. One challenge concerns the frequent lack of an intervention theory and conceptual clarity. In this regard, a number of frameworks exist that guide the development of public health interventions based on available evidence (e.g., the 6SQuID framework includes six crucial steps such as “identifying modifiable causal factors”; “deciding on mechanisms of change”) (27). Based on their review, Verschueren et al. (28) proposed a framework for theory-driven and evidence-based development of G4H that is comprised of five distinct stages, each including several elements: (1) scientific foundations (e.g., target audience, outcome objectives, theoretical basis), (2) design foundations (e.g., game mechanics, design requirements), (3) development (e.g., genre, results, content), (4) validation (i.e., clinical piloting), and (5) implementation (e.g., dissemination, rollout). However, most COVID-19 related games are based on global or national recommendations (e.g., on individual protective behaviors), but do not describe change mechanisms, their causal relation and how these can lead to the desired change (e.g., health knowledge or behavior). This also includes a lack of description of how game elements used relate to each other and contribute to the achievement of the intervention goals [a requirement that is highlighted by Verschueren et al. (28)]. Against this background, the poorly defined and synonymous use of the concepts “serious games” and “gamification” is not surprising. At a first glance, both concept share some communalities as they aim to promote health-related learning and behavioral change through increasing and maintaining user's motivation. However, while gamifications integrate game elements into non-game activities, serious games integrate educational content directly into the games, making the latter approach more difficult and complex, but also more promising to lasting effects. But differential effects of both approaches have yet to be examined, leaving it unclear which strategy is most appropriate for which purposes and target groups. Another challenge concerns the appeal and attractiveness of games in the context of the COVID-19 pandemic. G4H in particular compete with professional entertainment games or other forms of media (e.g., streaming, social media), making it necessary to find ways to capture user's interest. An appealing presentation has been identified as one of six factors of fun in action video games (29). However, this is countered by limited financial and human resources, which hardly allow to keep up with the high budget of commercial game companies. This might result in lower acceptance and user experience that limit usage and negatively affect effectiveness. But attractiveness is not only limited to the design but also the use of game mechanics and patterns. As mentioned earlier, the use of typical game elements (e.g., leader boards, points, badges) is often not justified, not balanced, and not tailored to the target group, leading to an “old” criticism that health-related messages are just sugar-coated that wear out quickly, i.e., become less interesting after a short while (“chocolate covered broccoli”). A positive example has been presented by Patel et al. who examined a gamification intervention to promote physical activity among economically disadvantaged adults (30). Findings provide evidence for sustained positive effects on physical activity when participants were able to self-choose goals and implement them directly. Finally, a third major challenge is the lack of evidence on the sustainability of effects and the factors that contribute to sustainability. As emphasized by Kermavnar and Desmet (15), only a few COVID-19 related G4H have been evaluated, often with small sample sizes and a focus on determinants of health behaviors such as knowledge or attitudes. In their evaluation of the game “COVID-19–Did You Know?”, Gaspar et al. (24) used google analytics to analyze users' correct and incorrect answer rates to various questions differentiated by topic. Findings indicate a significant reduction of error rates for questions around the topic “mask” over an 8-week period, but not for other topics. However, data were not based on a within-subjects design and did not include a control group. In another evaluation, Basol et al. (31) examined the efficacy of the 5-min browser game “Go Viral!” that aimed to improve the ability to identify manipulation techniques commonly used in COVID-19 misinformation. Based on a three-arm randomized controlled trial (RCT) an increase in perceived manipulativeness of misinformation and an improvement of the perceived ability to detect misinformation could be found immediately and 1 week after playing the “Go Viral!” intervention. Similarly, Suppan et al. (32) conducted a RCT to examine the effects of the “Escape COVID-19” quiz game on nursing home personnel's infection prevention and control (IPC) practices. Findings show a higher intention to change IPC practices for the intervention group immediately after playing the game, while actual behavior was not tested. Most evaluations are based on short follow-up periods, which makes it impossible to draw conclusions about the stability and sustainability of the effects. As discussed elsewhere, the transfer of games effects exclusively played in the virtual world to the real-world behavior is rarely considered. This applies to situations designed to be similar to the player's real world (first class transfer), but to an even greater extent to new situations (second class transfer) (33, 34). Overall, this makes it difficult to assess the “real-world” impact of such game-based interventions.

## 4. User involvement: The unused power of the crowd

There is widespread agreement in public health that those for which an intervention is intended should also be involved in its development and implementation. User involvement does not only contribute in better quality of health services and products but can also be regarded as a health promoting intervention itself resulting in better health outcomes (35). Even though we did not conduct a systematic review, we could hardly find any systematic approaches to user involvement in G4H on COVID-19. Often, user input and feedback are limited to pilot or feasibility studies, where aspects of user experience and usability were assessed (36, 37). One reason for this may be that, given the acute threat posed by the rapid spread of SARS-CoV-2, there was an urgent need for prompt public health actions, which conflicted with extensive (i.e., time consuming) user involvement. However, the problem appears to exist independently of the COVID-19 pandemic. A review of studies on serious games for healthcare professional's education showed that less than half of these games (21 of 45) with publications between 2000 and 2017 reported any form of user involvement (38). Only two of them involved users at the onset of the design stage, while in six games users were involved in later design stages and in another 12 games user involvement was limited to prototype testing. So obviously there is a high need for intensive user involvement that goes beyond sporadic consultancy or user testing. However, the conditions under which user involvement can foster game effectiveness are still unclear. In their review and meta-analysis, Desmet et al. (39) found that participatory design was associated with lower game effectiveness on behavior and self-efficacy with higher effectiveness for user as informant strategies and for the participatory design of game dynamics, levels, and game challenge. User involvement should therefore always be planned in such a way that the users have sufficient resources (e.g., professional support) and are not overburdened throughout the participatory process.

In addition to user's participation in the development and implementation, games can also be used as a tool allowing its users to contribute to research purposes. Active engagement of the general public or of population groups in research activities has been defined as “citizen science” that gained increasing attention in public health during the last years. The range of volunteer research activities spans from the provision of computing power (*crowd sourcing*), collecting, analyzing, and interpreting data after learning basic skills (*distributed intelligence*) to co-defining research questions and designs (*participatory science*) (40). Particularly in the case of large data sets, volunteer researchers can make an important contribution not only for prioritization of issues, but also in solving complex problems that would otherwise take long periods of time due to capacity limitations. There are now several citizen science games specifically designed to address health-related research goals. “Foldit” is a 3D folding puzzle where players are challenged to fold proteins into compact designs to discover the structure of a monkey HIV virus (41). “Borderlands 3” is a current example of integrating research issues into entertainment games. Entitled as “Borderland Science,” a mini game has been incorporated in the main game that presents players with simple block puzzles based on strands of DNA. By solving these puzzles, players help to map and compare the microbes contained therein (42). Another mini game within the open-world Massively Multiplayer Online Role-Playing Game (MMORPG) “Eve Online” motivates its users to analyse the blood of individuals infected with COVID-19 for disease markers by finding and marking cell clusters (43). The number of cell clusters marked by the players corresponds to 471 years that researchers would have to spend on this task. Citizen science games leverage social engagement in health issues and could make a significant contribution to solving current and future (health) crises, e.g., in the study of climate change and its consequences for health.

## 5. Concluding remarks

The COVID-19 pandemic, which has been ongoing for almost 3 years now, highlights the importance of preparing for and appropriately dealing with health crises and emergency situations. Although G4H are increasingly used in public health research and practice, they only played a minor role during the pandemic. One reason for this may be the need for quick solutions given the rapid spread of the virus, which hindered time-consuming development and implementation of G4H. In this paper, we argued for greater collaboration between public health stakeholders and commercial game makers to reach large audiences, especially those who often cannot be adequately reached through traditional health communication efforts (e.g., racial, ethnic, and other minorized populations experiencing health disparities). This could include health messages distributed *via* the characters and plots of popular entertainment games. In addition, well-established games could motivate the gaming community to contribute to solving research problems in a fun way, e.g., by integrating scientific mini-games for big (health) data analysis. Game reuse and flexible game frameworks are two other strategies for targeting the use of games in times of crises. While the first one uses well-known games or game genres and transfer that to the health context, game frameworks provide health-related stakeholders the possibility to tailor a G4H to their specific needs (10).

In addition to interventions designed to respond immediately to crises and reach large populations, we have identified several challenges that public health research will need to address in the future. This includes strengthening the underlying intervention theory of G4H by, for example, justifying the use of the game content and game mechanics employed in terms of their intended effects. As mentioned before, an explicit differentiation of the concepts serious games and gamification is needed to enable useful game-based scenarios and to create a sound evidence-base in both fields. Regarding crisis management, serious games seem to be more useful to address certain crisis related topics. The use of gamification is a more universal approach and can be used to support the learning process in teaching crisis related issues (like modified hygiene procedures) or for health-related behavior change interventions (like promoting adapted behavior in case of hazardous environments). As entertainment media compete for the favor of their target audience, health-related games must also keep pace with commercial offerings and be attractive. This requires sufficient resources and, in particular, collaboration with User Experience designers as early as possible in the planning phase. As emphasized, the end users for whom games are being developed should also be involved, and further research is needed to determine the right level, intensity, and conditions of involvement needed. Finally, we strongly advocate improving the evidence base using appropriate study designs that can link data on usage (e.g., duration, intensity) with data on (long-term) effectiveness. This will make it possible to generate evidence on the conditions necessary of game effectiveness. As mentioned above, further studies are needed that focus more closely on the effects of G4H interventions, particularly among vulnerable and disadvantaged populations (14).

## Data availability statement

The original contributions presented in the study are included in the article/supplementary material, further inquiries can be directed to the corresponding author.

## Author contributions

KD wrote the first version of the manuscript. FF, DT, KW, and TM contributed substantially to manuscript revision. All authors read and approved the final version of the paper.
